# Fasting Glucose Variability as a Risk Indicator for End-Stage Kidney Disease in Patients with Diabetes: A Nationwide Population-Based Study

**DOI:** 10.3390/jcm10245948

**Published:** 2021-12-18

**Authors:** Da Young Lee, Jaeyoung Kim, Sanghyun Park, So Young Park, Ji Hee Yu, Ji A. Seo, Nam Hoon Kim, Hye Jin Yoo, Sin Gon Kim, Kyung Mook Choi, Sei Hyun Baik, Kyungdo Han, Nan Hee Kim

**Affiliations:** 1 Division of Endocrinology and Metabolism, Department of Internal Medicine, Korea University College of Medicine, Seoul 02841, Korea; ddkristin412@gmail.com (D.Y.L.); psyou0623@gmail.com (S.Y.P.); dniw99@gmail.com (J.H.Y.); seojia@korea.ac.kr (J.A.S.); pourlife@naver.com (N.H.K.); deisy21@naver.com (H.J.Y.); k50367@korea.ac.kr (S.G.K.); medica7@gmail.com (K.M.C.); 103hyun@gmail.com (S.H.B.); 2 Research Institute for Skin Image, Korea University College of Medicine, Seoul 08308, Korea; jaykim830@gmail.com; 3 Core Research & Development Center, Korea University Ansan Hospital, Ansan 15355, Korea; 4 Division of Endocrinology and Metabolism, Department of Internal Medicine, Seoul St. Mary’s Hospital, College of Medicine, The Catholic University of Korea, Seoul 06591, Korea; ujk8774@naver.com; 5 Department of Statistics and Actuarial Science, Soongsil University, Seoul 06978, Korea; 6 BK21 FOUR R&E Center for Learning Health Systems, Korea University, Seoul 02841, Korea

**Keywords:** diabetes mellitus, glucose variability, end-stage kidney disease, Korean National Health Insurance Corporation

## Abstract

Given the fact that diabetes remains a leading cause of end-stage kidney disease (ESKD), multi-aspect approaches anticipating the risk for ESKD and timely correction are crucial. We investigated whether fasting glucose variability (FGV) could anticipate the development of ESKD and identify the population prone to the harmful effects of GV. We included 777,192 Koreans with diabetes who had undergone health examinations more than three times in 2005–2010. We evaluated the risk of the first diagnosis of ESKD until 2017, according to the quartile of variability independent of the mean (VIM) of FG using multivariate-adjusted Cox proportional hazards analyses. During the 8-year follow-up, a total of 7290 incidents of ESKD were found. Subjects in the FG VIM quartile 4 had a 27% higher risk for ESKD compared to quartile 1, with adjustment for cardiovascular risk factors and the characteristics of diabetes. This effect was more distinct in patients aged < 65 years; those with a long duration of diabetes; the presence of hypertension or dyslipidemia; and prescribed angiotensin-converting enzyme inhibitors, metformin, sulfonylurea, α-glucosidase inhibitors, and insulin. In contrast, the relationship between baseline FG status and ESKD risk showed a U-shaped association. FGV is an independent risk factor for kidney failure regardless of FG.

## 1. Introduction

Diabetes remains a leading cause of end-stage kidney disease (ESKD) globally and accounts for 35–50% of these cases [1].

Although several medications, such as sodium-glucose cotransporter 2 inhibitors (SGLT2 inhibitors), angiotensin-converting enzyme inhibitors (ACE inhibitors), and angiotensin-receptor blockers (ARBs), have some protective mechanism against deterioration of renal function, their prevention capacity for ESKD is only 22–40% [2,3]. Therefore, to reduce the burden of ESKD, multi-aspect approaches exploring new biomarkers for anticipating the risk for ESKD and timely correction are crucial in patients with diabetes.

The variability of cardio-metabolic parameters has been an interesting issue because of its predictive value for numerous clinical outcomes [4,5]. Glucose variability (GV) consists of short-term, intraday GV derived from the continuous glucose monitoring system and long-term fasting glucose (FG) variability over several months to years, reflecting the stability of the medication’s effect, adherence, and residual insulin secretion [6]. Several studies have reported that high GV is associated with an increased risk of diabetic vascular complications [7,8], heart failure [9], and poor prognosis for acute lung diseases [10].

Regarding kidney outcomes, long-term variability in comprehensive cardio-metabolic risk factors showed a positive association with the future risk of ESKD in the general population, but not in diabetes [4,11]. Furthermore, in patients with diabetes, most evidence adopted glycated hemoglobin (HbA1c) variability rather than GV, and study outcomes were the development of macroalbuminuria or kidney function decline, rather than the development of ESKD [12,13,14,15]. This is attributed to the lower incidence rates of ESKD compared to other diabetic vascular complications, such as cardiovascular disease (CVD) [16]. To overcome this limitation, large-scale epidemiologic studies are essential to explore ESKD outcomes.

Therefore, we investigated whether FGV could predict the risk of ESKD using nationally representative population-based cohort data in Korea. We also compared the impact of FGV with FG on future ESKD risk and verified the specific population prone to the detrimental effect of higher FGV.

## 2. Materials and Methods

### 2.1. Study Design and Subjects

This was a retrospective observational study (Figures S1 and S2). We extracted the data of the participants who had undergone health examinations supported by the National Health Insurance Corporation (NHIC) at least twice from 2005 to 2008, and simultaneously at least once between 1 January 2009 and 31 December 2010 (referred to as “baseline exam”). That is, the study subjects underwent at least three health examinations during the five years between 2005 and 2010 (referred to as the FGV assessment period). Among them, we excluded 16,736,363 participants without diabetes, aged < 40 years; those with previous histories of ESKD and missing data in the inclusion criteria; and those who were diagnosed with ESKD within one year after baseline. A total of 777,192 participants were included in the study.

The NHIC is a nationally operating health insurance system in Korea and covers approximately 97% of Koreans. The NHIC database contains eligibility information; health examination results, including questionnaires on lifestyle; and a medical care institution database [17,18]. Enrollees of the NHIC are encouraged to perform a standardized medical examination annually or biannually. Information about medical treatments was recognized by the medical bills charged by healthcare providers with the International Classification of Diseases, 10th Revision (ICD-10).

This research was approved by the NHIC and the Institutional Review Board of the Korea University Ansan Hospital (2019AS0138) and followed the Helsinki Declaration of 1975.

### 2.2. Anthropometric and Laboratory Measurements

Demographic characteristics, lifestyle habits, and medical history were identified using questionnaires during medical examinations. Alcohol consumption was categorized as near abstinence, moderate (<30 g/day), or severe (≥30 g/day). Smoking history was stratified into never, ex-, and current smokers. Regular exercise was defined as >30 min of moderate-intensity exercise or >20 min of vigorous-intensity exercise ≥1 per week [19].

Body mass index was calculated as weight (kg) divided by the square of height (m). Blood pressure (BP) was checked after ≥5 min of rest.

Venous blood sampling was conducted in the morning after an overnight fast of ≥8 h to measure the concentrations of hemoglobin, plasma glucose, creatinine, high-density lipoprotein cholesterol, low-density lipoprotein cholesterol, triglycerides, and total cholesterol.

Midstream urine samples were collected to measure urine protein using a urine dipstick with the following grades: absent, trace (±), 1+, 2+, 3+, and 4+, which correspond to the amount of urine protein of undetectable, 10, 30, 100, 300, and 1000 mg/dL, respectively [4].

Quality control of laboratory tests was performed, followed by the Korean Association of Laboratory Quality Control.

### 2.3. Definition of Glucose Variability

Using FG concentrations measured at least three times during the five years prior to and including the baseline, the variability independent of the mean (VIM) of FG was calculated as a primary variability indicator (Figure S2). The equation is as follows:

$$\mathrm{VIM}=100\times \frac{\mathrm{SD}}{{\mathrm{mean}}^{\beta }}$$

Standard deviation (SD), coefficient of variation (CV, SD/mean), and average real variability (ARV) were estimated [20].

$$\mathrm{ARV}=\frac{1}{n-1}\sum_{k=1}^{n-1}\times \left|{\mathrm{BP}}_{k+1}-{\mathrm{BP}}_{K}\right|$$

where n is the number of FG measurements, and k ranges from 1 to n − 1.

### 2.4. Operational Definition of Diseases

Diabetes was defined as a fasting plasma glucose level ≥ 126 mg/dL or at least one prescription of glucose-lowering medicine (GLM) per year with ICD-10 codes E10–14. We defined type 1 diabetes in patients if they had both an ICD-10 code E10 and at least one prescription history of insulin, while the remaining patients were referred to as having type 2 diabetes.

The study outcome was a new diagnosis of ESKD, identified by the initiation of renal replacement therapy or kidney transplantation under ICD-10 codes N18–19, Z49, Z90, Z94, or Z99.2 [21]. Because dialysis is reimbursed when registered in Korea, we could discern all cases of renal replacement therapy under the claim codes for peritoneal dialysis (O7071-O7075 or V003), hemodialysis (O7011-O7020 or V001), and kidney transplantation (R3280) [21]. We excluded acute renal failure events, which were defined as individuals with transient renal replacement therapy or continuous renal replacement therapy without a previous history of CKD. Deceased cases, identified by the nationwide death certificate data of the Korea National Statistical Office, were censored at the time of their death. The follow-up period was calculated from the time interval between the baseline exam and incident ESKD, date of death, or 31 December 2017, whichever came first (Figure S2).

Hypertension was defined as systolic BP ≥ 140 mmHg, diastolic BP ≥ 90 mmHg, or at least one prescription of antihypertensive drugs per year under ICD-10 codes I10–I15. The presence of malignancy was defined by registration in the Korea Central Cancer Registry with ICD-10 C00–C96 before the baseline examination. Low-income status was defined as the lowest 20% income identified by the amount of health insurance premium or eligibility as medical care [17,18]. Dyslipidemia was determined by total cholesterol concentration ≥6.21 mmol/L or at least one prescription of antihyperlipidemic medications under ICD-10 code E78. The estimated glomerular filtration rate (eGFR) < 60 mL/min/1.73 m^2^, estimated by the Modification of Diet in Renal Disease formula [22], was stratified according to the presence of chronic kidney disease (CKD) [23].

The prescription of ACE inhibitors or ARBs, oral GLM among metformin, sulfonylurea, meglitinide, thiazolidinedione, inhibitors of dipeptidyl peptidase 4 (DPP-4 inhibitors), α-glucosidase inhibitor (AGI), and insulin in the 12 months before baseline was identified. History of heart disease or stroke was estimated using self-reports.

### 2.5. Statistical Analysis

Data are shown as mean ± SD, median (interquartile range), or number (%). After stratifying the subjects according to the FG VIM quartile, we compared baseline features using chi-squared tests and analysis of variance for continuous variables. Triglyceride concentrations were log-transformed for the analysis.

Multivariable regression analyses were conducted using the Cox proportional hazards model to estimate the time-dependent risk of ESKD according to FG VIM quartiles, with quartile 1 as the reference group. In model 1, age, sex, body mass index, alcohol drinking, smoking, exercise, presence of CKD, hypertension, dyslipidemia, and low-income status were adjusted. In model 2, the duration of diabetes as continuous variable, insulin prescription, the number of classes of oral GLM during 12 months prior to baseline exam, mean FG measured for the five years preceding the baseline exam, and the number of exams were additionally adjusted. To evaluate the change in significance according to the cutoff value of VIM, we further divided the study population into deciles and reiterated the above-mentioned regression analysis with decile 1 as a reference. In addition, we explored whether the main findings would change after replacing the parameters of FGV with SD, CV, and ARV instead of VIM.

For subgroup analyses, we determined the hazard ratios (HRs) and 95% confidence intervals (CIs) of FG VIM quartile 4 versus quartile 1–3 for ESKD after dividing the subjects according to clinically relevant factors and the characteristics of diabetes. Regression analysis was performed using the same adjustment strategy.

To evaluate the association of a single FG concentration with the risk of ESKD, we repeated the analysis according to baseline FG concentration, with 100–119 mg/dL as a reference group. The mean FG was excluded as a confounder in this analysis.

We found a variable inflation factor for all covariates of less than 2.0, and there was no multicollinearity in the covariates. Statistical analysis was performed using SAS version 9.3 (SAS Institute Inc., Cary, NC, USA). Statistical significance was set at *p* < 0.05.

## 3. Results

Compared with participants in the FG VIM quartile 1, those in the FG VIM quartile 4 were younger, had a higher proportion of males, were current smokers, and had higher fasting glucose and triglyceride levels (Table 1). Among comorbidities, they had more CKD but less hypertension, dyslipidemia, ischemic heart disease, and stroke. In the case of the characteristics of diabetes, people in FG VIM quartile 4 had a higher proportion of insulin users, individuals prescribed with ≥2 GLM during one year before baseline, and those with a duration of diabetes of at least five years.

During 8.0 (7.4–8.4) years of median (interquartile range) follow-up period, a total of 7290 cases of ESKD were identified (Table 2). Age- and sex-adjusted HRs for ESKD serially increased as the FG VIM quartile increased. In model 2, the HR (95% CI) for ESKD of participants in FG VIM quartile 4 was 1.27 (1.19–1.36), with adjustment for clinically relevant factors, duration of diabetes, history of CKD, mean FG, and the number of exams. When the participants were divided into deciles in more detail, significantly higher risks for ESKD were found in D9 and D10 with a cutoff value of D9 of 34.4 (Table S1). A similar association was observed when FGV parameters were changed to SD, CV, and ARV (Table S2).

In subgroup analyses, increased risk for ESKD in VIM quartile 4 versus quartile 1–3 was more evident in individuals aged 40–64 years, with a prescription history of ACE inhibitors or ARBs, hypertension, and dyslipidemia (Table 3). Among the various characteristics of diabetes, the impact of higher FGV was more distinct in patients with a long duration of diabetes and the prescription of metformin, sulfonylurea, AGI, and insulin (Table 4).

On the other hand, baseline FG levels showed a U-shaped association with the risk of ESKD (Table S3). Compared to participants whose FG concentrations were in the range of 100–119 mg/dL, individuals with FG < 100 mg/dL or ≥180 mg/dL had a higher risk of ESKD.

## 4. Discussion

### 4.1. Significant Findings of the Present Study

These results confirmed the hypothesis that FGV is significantly associated with an increased risk of ESKD among patients with diabetes. The risk for ESKD was 27% higher in the group with the highest FGV than in the lowest FGV group. The predictive value of high FGV on the incident ESKD was more prominent in patients with young age; hypertension; dyslipidemia; a long duration of diabetes; and who were treated with ACE inhibitors or ARBs, metformin, sulfonylurea, AGI, and insulin. In contrast, the association between FG and the risk of ESKD was U-shaped.

### 4.2. Kidney Outcomes and Long-Term Glucose Variability

Most previous studies have chosen HbA1c variability rather than FG variability for glucose variability assessment, and their study outcomes were renal function decline or development of albuminuria, not ESKD [17,18,19,21]. In the Action in Diabetes and Vascular Disease: Preterax and Diamicron MR Controlled Evaluation (ADVANCE) trial, SD of FG over 24 months exhibited a positive association with the risk of nephropathy combined with retinopathy [8]. Recently, a meta-analysis of three well-known clinical trials, the U.K. Prospective Diabetes Study, the Action to Control Cardiovascular Risk in Diabetes trial, and the Veteran Affairs Diabetes Trial, showed that FGV was associated with a 30–40% increase in the risk of incident moderate to severe nephropathy, defined by eGFR < 45 mL/min/1.73 m^2^ [24].

Only one study has evaluated the impact of FGV on the development of ESKD using a population-based study [25,26]. The Taiwan Diabetes Study reported that FG-CV and HbA1c-CV could predict the development of diabetic nephropathy [27] and ESKD [28] in patients with type 2 diabetes. In the present study, compared to the previous one, we included more patients with diabetes (*n* = 777,192 vs. 31,841) and calculated the FGV for a longer period (5 vs. 1 year). Although ESKD is a hard outcome of diabetic renal complications, it is hard to study ESKD as an outcome due to the lower incidence. The incidence rate of ESKD in 2018 was 374.7 cases per million [16], lower than that of CVD, at 8980 cases per million in 2017 [29]. To overcome this limitation, a large population-based study is necessary. Because the NHIC entirely operates the health insurance system in Korea, we could use almost all Koreans with diabetes and subsequently obtained 777,192 individuals eligible for this study, making it possible to perform a more detailed subgroup analysis.

In patients with diabetes, oscillation in the FG level during a long follow-up period might reflect poor self-care, overall poor compliance, and suboptimal strategy for GLMs [30]. Because HbA1c is the average plasma glucose during 2–3 months, HbA1c variability implies a change in glycemic status rather than glucose fluctuation itself. In other words, FG might be better at capturing real-time glucose variations than HbA1c levels [7]. Therefore, FGV in our study was derived from yearly or biannually measured FG levels over five years, allowing for a comprehensive evaluation of a patient over a long period of time. In addition, this simple strategy for estimating FGV could be helpful for public health policy makers to select high-risk populations and support active prevention.

On the other hand, a high risk for ESKD was observed in individuals whose baseline FG levels were <100mg/dL or ≥180 mg/dL. These findings were consistent with another nationwide cohort study of Koreans with diabetes using GLMs [31], suggesting that intensive glucose control might not necessarily diminish the progression of established diabetic kidney disease.

### 4.3. Interpretation for the Impact of Glucose Variability

There is little data available to explain the mechanism linking glucose variability and ESKD risk directly. Cha et al. demonstrated the negative association of plasma adiponectin and glypican-4 levels with eGFR and positive association with urinary albumin levels [32]. The findings that transient glucose spikes could induce oxidative stress and impair endothelial function more than sustained hyperglycemia [33,34] and that glomerular permeability, mesangial lipid accumulation, and collagen synthesis are increased after intermittent exposure to high glucose levels [25,26] could be a pathophysiologic explanation of this association.

The results of the subgroup analysis provide a chance to identify the population more vulnerable to FGV (Table 3). It is possible that individuals with a long duration of diabetes are sensitive to oxidative stress because their enzymatic antioxidant defense systems are less efficient [29,35]. The presence of hypertension or dyslipidemia itself is an already proven risk factor for ESKD [36]. Its significant interaction with the harmful effect of high FGV on the risk of ESKD suggests a synergic relationship. 

Interestingly, a significant effect of FGV was not observed in individuals aged ≥ 65 years. This may be due to the competing risk of death in patients with diabetic ESKD [37]. A Finnish nationwide cohort study showed that the cumulative risk of ESKD decreased with increasing age [38]. At the same time, mortality increased among the older age groups, with a 100-fold higher incidence of death than the ESKD cases throughout the 20-year-of follow-up [38]. Therefore, we theorize that the deceased cases ahead of ESKD might diminish the ESKD cases, weakening the effect of FGV. 

The valid interaction with the prescription of ACE inhibitors or ARB, metformin, sulfonylurea, AGI, and insulin should be interpreted cautiously. There have been no previous studies exploring the interaction between GLM and the impact of FGV on ESKD, but only showed that sulfonylurea increases the glucose variability [39], whereas DPP-4 inhibitors and degludec reduced it [40,41]. Subjects with higher FGV might be treated with more GLMs due to their clinical condition. If they were not prescribed more GLMs, their FGV would be higher, and the association with ESKD risk might be stronger than in the present study. 

SGLT2 inhibitors and glucagon-like peptide-1 receptor agonists, which have been known to prevent CKD progression, have been reimbursable for patients with diabetes in Korea since 2014 and 2015, respectively [2,3]. Because the prescriptions of these GLMs were negligible during the glucose variability assessment period (2005–2010), their impact on the incidence of ESKD until 2017 was expected to be minimal. 

### 4.4. Parameters for Estimating Glycemic Variability

There is no consensus on a standardized index for glucose variability with distinct characteristics [42]. SD refers to the dispersion of measurements around the mean, and CV reflects a standardized variation that provides direct comparison among study groups. ARV is the average of the absolute differences of successive measurements and might be a reliable index for time series variability [20,43]. However, we chose VIM as the primary parameter of FGV because VIM is a measure of variability designed not to correlate with mean levels which is appropriate for the purpose of this study [44]. SD, CV, and ASV are partially dependent on mean despite of adjustment for mean value [45]. When we analyzed SD or CV again, a similar trend was observed (Table S2).

### 4.5. Limitations

This nationwide population-based study clearly showed the influence of long-term FG variability on incident ESKD with a long-term follow-up period. The 5-year FGV levels used in the present study were much longer than those used in previous studies. However, several limitations of this study should be considered.

First, given that we extracted study subjects according to the times of health check-ups to calculate long-term glucose variability, those with healthier lifestyle and slightly elevated glucose concentrations could be included, which might be a source of selection bias. Moreover, it is not available for complete information of hypoglycemia events. Second, postprandial glucose, HbA1c, serum c-peptide, and autoantibody levels were not included in this database. To enhance the accuracy of diagnosis of diabetes and subtype, we used ICD-10 codes with prescription histories of GLM and FG levels. Although we could not use HbA1c variability, the variability of FG was a stronger predictor of microvascular and macrovascular events than HbA1c variability in the ADVANCE trial [8]. Third, health examinations provided by the NHIC measure only dipstick proteinuria, not urine albuminuria. Finally, given the retrospective design of this study, reverse causation and undetected exposure of the risk factors of ESKD were possible [46]. We excluded incident ESKD cases developed one year after the baseline to minimize this issue. Additionally, the fasting period was not standardized fasting period could influence the FG levels.

Despite those limitations, a large-sized population-based cohort study covering almost entire Koreans is still the most suitable design for investigating rare outcomes such as ESKD possible [46].

## 5. Conclusions

This large-scale nationwide population-based study demonstrated that FG variability was independently associated with an increased risk of ESKD among patients with diabetes, especially in those with young age, long duration of diabetes, and comorbidities who need more GLM and RAS inhibitors. These findings highlight that reducing FGV is a vital strategy to reduce the incidence of ESKD in diabetes, especially in high-risk populations.

## Figures and Tables

**Table 1:** Baseline characteristics of the study subjects according to quartiles of fasting glucose variability ^a^.

Characteristics	VIM Q1 (*n* = 194,302)	VIM Q2 (*n* = 194,291)	VIM Q3 (*n* = 194,301)	VIM Q4 (*n* = 194,298)	*p*-Value
Age (years)	61.2 ± 9.8	60.2 ± 10.0	59.7 ± 10.2	59.4 ± 10.5	<0.001
Sex, male (%)	109,509 (56.4)	116,074 (59.7)	120,274 (61.9)	125,355 (64.5)	<0.001
BMI (kg/m^2^)	24.7 ± 3	24.9 ± 3.1	24.9 ± 3.1	24.8 ± 3.2	<0.001
Systolic BP (mmHg)	128.3 ± 15.2	128.7 ± 15.2	128.8 ± 15.3	128.5 ± 15.3	<0.001
Fasting glucose (mg/dL)	125.0 ± 33.9	130.1 ± 35.5	135.7 ± 39.0	146.0 ± 53.4	<0.001
Total cholesterol (mg/dL)	193.6 ± 39.1	194.9 ± 39.9	195.6 ± 40.7	194.4 ± 41.5	<0.001
Triglyceride (mg/dL)	132.9 (132.5–133.2)	138.4 (138.1–138.8)	143(142.6–143.4)	146.3 (146.0–146.7)	<0.001
HDL-C (mg/dL)	52.7 ± 22.8	52.3 ± 21.5	52 ± 21.8	51.5 ± 21.3	<0.001
LDL-C (mg/dL)	111.6 ± 43.0	111.6 ± 42.7	111.2 ± 43.4	109.5 ± 44.5	<0.001
GLU_VIM (%)	8.2 ± 3	16.6 ± 2.2	25.5 ± 3	43.5 ± 11.1	<0.001
GLU_SD (mg/dL)	8.1 ± 5.3	16.8 ± 8.5	26.7 ± 13.1	49.0 ± 25.2	<0.001
GLU_CV (%)	6.2 ± 2.6	12.7 ± 2.9	19.9 ± 4.3	35 ± 11.2	<0.001
GLU_ARV (mg/dL)	10 ± 7.2	20.3 ±11.9	31.6 ± 18.3	56.5 ± 34.3	<0.001
Current smoker (%)	31,644 (16.3)	36,873 (19.0)	42,614 (21.9)	50,137 (25.8)	<0.001
Heavy drinking (%)	12,395 (6.4)	13,844 (7.1)	14,670 (7.6)	14,289 (7.4)	<0.001
Regular exercise (%)	49,893 (25.7)	48,442 (24.9)	46,317 (23.8)	43,246 (22.3)	<0.001
eGFR (mL/minute/1.73 m^2^)	79.6 (68.5–92.6)	79.9 (68.5–92.9)	80.1 (68.5–93.3)	79.6 (67.7–92.9)	<0.001
Chronic kidney disease (%) ^b^	23,041 (11.9)	22,930 (11.8)	23,569 (12.1)	26,133 (13.5)	<0.001
Dipstick proteinuria (%)					<0.001
Absence (%)	178,444 (91.8)	177,043 (91.1)	175,837 (90.5)	173,973 (89.5)	
Trace (%)	6065 (3.1)	6380 (3.3)	6777 (3.5)	6723 (3.5)	
1 + (%)	5939 (3.1)	6580 (3.4)	6999 (3.6)	7742 (4)	
2 + (%)	2841 (1.5)	3149 (1.6)	3450 (1.8)	4205 (2.2)	
3 + (%)	841 (0.4)	924 (0.5)	1064 (0.6)	1378 (0.7)	
4 + (%)	172 (0.1)	215 (0.1)	174 (0.1)	277 (0.1)	
Comorbidities					
Hypertension (%)	119,605 (61.6)	117,761 (60.6)	115,704 (59.6)	112,881 (58.1)	<0.001
Dyslipidemia (%)	102,627 (52.8)	98,666 (50.8)	95,100 (48.9)	90,667 (46.7)	<0.001
IHD (%)	28,614 (14.7)	26,445 (13.6)	24,879 (12.8)	23,758 (12.2)	<0.001
Stroke (%)	10,979 (5.7)	10,286 (5.3)	9961 (5.1)	9996 (5.1)	<0.001
Income (lower 20%, %)	34,931 (18.0)	36,804 (18.9)	39,098 (20.1)	43,447 (22.4)	<0.001
ACE inhibitors or ARBs (%)	71,197 (36.6)	69,355 (35.7)	67,950 (35.0)	67,800 (34.9)	<0.001
Oral GLM					
Metformin	72,551 (37.3)	75,633 (38.9)	79,615 (41.0)	85,739 (44.1)	<0.001
Sulfonylurea	70,505 (36.3)	76,924 (39.6)	84,825 (43.7)	92,837 (47.8)	<0.001
Meglitinide	3960 (2)	4286 (2.2)	4821 (2.5)	5950 (3.1)	<0.001
Thiazolidinedione	11,624 (6)	12,466 (6.4)	13,402 (6.9)	14,708 (7.6)	<0.001
DPP-4 inhibitor	7602 (3.9)	7871 (4.1)	8300 (4.3)	8531 (4.4)	<0.001
a-Glucosidase inhibitor	18,941 (9.8)	21,134 (10.9)	24,274 (12.5)	28,984 (14.9)	<0.001
Number of oral GLM					<0.001
0	96,962 (49.9)	93,619 (48.2)	88,878 (45.7)	82,779 (42.6)	
1	34,341 (17.7)	31,949 (16.4)	29,574 (15.2)	26,813 (13.8)	
2	42,096 (21.7)	44,622 (23.0)	47,759 (24.6)	51,446 (26.5)	
3	17,310 (8.9)	19,723 (10.2)	22,828 (11.8)	26,763 (13.8)	
≥4	3593 (1.9)	4378 (2.3)	5262 (2.7)	6497 (3.3)	
Insulin	8125 (4.2)	9515 (4.9)	11,928 (6.1)	19,582 (10.1)	<0.001
Duration of diabetes	2.7 ± 3.1	2.8 ± 3.1	3 ± 3.2	3.3 ± 3.2	<0.001
≥5 years (%)	56,944 (29.3)	59,454 (30.6)	63,309 (32.6)	68,451 (35.2)	<0.001
Type 1 diabetes (%)	1274 (0.7)	1537 (0.8)	2106 (1.1)	4153 (2.1)	<0.001
Number of exams					<0.001
3	167,018 (86.0)	152,379 (78.4)	146,220 (75.3)	142,455 (73.3)	
4	13,832 (7.1)	19,418 (10.0)	22,307 (11.5)	24,566 (12.6)	
5	13,452 (6.9)	22,494 (11.6)	25,774 (13.3)	27,277 (14)	
Time interval between adjacent exams (years)	1.87 (1.3–2.1)	1.8 (1.1–2.1)	1.76 (1.1–2.1)	1.71 (1.1–2.1)	<0.001

^a^ Q1: 0–12.7; Q2: 12.8–20.6; Q3: 20.7–31.2; Q4: ≥31.3. ^b^ Presence of chronic kidney disease represents estimated glomerular filtration rate < 60 mL/minute/1.73 m^2^. Data are presented as mean ± standard deviation, median (interquartile range), or number (%). One-way analysis of variance and the chi-squared test were used to compare the characteristics of the study subjects at baseline. Post hoc multiple comparison analysis was performed with Bonferroni correction, and triglyceride levels were log-transformed for analysis. *p*-values were <0.001 for all variables because of the large sample size. Abbreviations: VIM, variability independent of mean; BMI, body mass index; BP, blood pressure; HDL-C, high-density lipoprotein-cholesterol; LDL-C, low-density lipoprotein-cholesterol; SD, standard deviation; CV, coefficient of variation; ARV, average real variability; eGFR, estimated glomerular filtration rate; IHD, ischemic heart disease; ACE inhibitor, angiotensin-converting enzyme inhibitor; ARB, angiotensin-receptor blocker; GLM, glucose-lowering medicine; DPP-4 inhibitor, inhibitors of dipeptidyl peptidase 4; ICD-10, International Classification of Diseases, 10th Revision.

**Table 2:** Hazard ratios and 95% confidence intervals for the incidence of end-stage of kidney disease by quartiles of fasting glucose variability ^a^.

	Events (*n*)	Follow-Up Duration (Person-Years)	Incidence Rate (Per 1000 Person-Years)	Age- and Sex- Adjusted HR (95% CI)	Multivariate-Adjusted HR (95% CI)	
					Model 1	Model 2
Q1 (*n* = 194,302)	1412	1,478,422.2	0.96	1 (Ref.)	1 (Ref.)	1 (Ref.)
Q2 (*n* = 194,291)	1487	1,483,681.0	1.00	1.07 (0.99–1.15)	1.05 (0.97–1.13)	0.99 (0.92–1.06)
Q3 (*n* = 194,301)	1721	1,482,829.3	1.16	1.25 (1.16–1.34)	1.21 (1.12–1.3)	1.03 (0.96–1.1)
Q4 (*n* = 194,298)	2670	1,468,254.3	1.82	1.96 (1.84–2.10)	1.79 (1.68–1.91)	1.27 (1.19–1.36)

^a^ Q1: 0–12.7; Q2: 12.8–20.5; Q3: 20.6–31.2; Q4: ≥31.3. Model 1 is adjusted for age, sex, body mass index, smoking, alcohol drinking, exercise, presence of chronic kidney disease, dyslipidemia, hypertension, and low-income status. Model 2 is the same as model 1, plus an adjustment for duration of diabetes as continuous variable, the number of classes of oral glucose-lowering medicine, the presence of prescription history of insulin, the mean of fasting glucose, and the number of exams.

**Table 3:** Subgroup analysis according to clinically relevant factors in the fasting glucose variability quartile 4 versus quartiles 1–3.

	IR per 1000	HR (95% CI)	*p* for Interaction
Age (years)			0.000
40–64 (*n* = 521,902)	1.50	1.36 (1.28–1.45)	
≥65 (*n* = 255,290)	2.61	1.14 (1.06–1.23)	
Sex			0.849
Male (*n* = 471,212)	2.02	1.26 (1.19–1.33)	
Female (*n* = 305,980)	1.46	1.27 (1.16–1.39)	
BMI			0.325
<25 kg/m^2^ (*n* = 425,481)	1.94	1.24 (1.16–1.32)	
≥25 kg/m^2^ (*n* = 351,711)	1.68	1.3 (1.2–1.4)	
Current smoking			0.215
No (*n* = 615,924)	1.88	1.28 (1.21–1.35)	
Yes (*n* = 161,268)	1.63	1.19 (1.08–1.32)	
Hypertension			0.004
No (*n* = 311,241)	0.51	1.05 (0.92–1.2)	
Yes (*n* = 465,951)	2.80	1.3 (1.23–1.37)	
ACE inhibitor or ARB			0.001
No (*n* = 500,890)	0.70	1.11 (1.01–1.21)	
Yes (*n* = 276,302)	3.99	1.33 (1.25–1.4)	
Chronic kidney disease			0.988
No (*n* = 681,519)	0.75	1.26 (1.17–1.36)	
Yes (*n*= 95,673)	9.33	1.26 (1.19–1.34)	
Dyslipidemia			0.035
No (*n* = 390,132)	1.15	1.18 (1.09–1.28)	
Yes (*n* = 387,060)	2.58	1.31 (1.23–1.39)	
Income lower 20%			0.636
No (*n* = 622,912)	1.79	1.27 (1.2–1.34)	
Yes (*n* = 154,280)	1.92	1.23 (1.12–1.37)	

Adjusted for age, sex, body mass index, smoking, alcohol drinking, exercise, presence of dyslipidemia, hypertension, chronic kidney disease, low-income status, duration of diabetes as continuous variable, the number of classes of oral glucose-lowering medicine, presence of prescription history of insulin, mean fasting, and the number of exams. Each variable used to stratify the participants was excluded from the adjustment.

**Table 4:** Subgroup analysis according to the characteristics of diabetes in the fasting glucose variability quartile 4 versus quartiles 1–3.

	IR per 1000	HR (95% CI)	*p* for Interaction
Baseline fasting glucose			0.305
<126 mg/dL (*n* = 349,855)	2.67	1.16 (1.08–1.25)	
≥126 mg/dL (*n* = 427,337)	1.34	1.23 (1.15–1.31)	
Duration of diabetes			<0001
<5 years (*n* = 529,034)	0.63	1.01 (0.92–1.11)	
≥5 years (*n* = 248,158)	4.11	1.38 (1.3–1.46)	
Type of diabetes			0.348
Type 2 diabetes (*n* = 768,122)	1.65	1.26 (1.19–1.32)	
Type 1 diabetes (*n* = 9070)	10.06	1.16 (0.98–1.36)	
Metformin			0.002
No (*n* = 463,634)	1.34	1.16 (1.08–1.25)	
Yes (*n* = 313,538)	2.43	1.35 (1.26–1.44)	
Sulfonylurea			0.011
No (*n* = 452,101)	1.24	1.16 (1.07–1.26)	
Yes (*n* = 325,091)	2.46	1.32 (1.25–1.41)	
Meglitinide			0.276
No (*n* = 758,175)	1.69	1.27 (1.21–1.34)	
Yes (*n* = 19,017)	5.99	1.16 (0.99–1.36)	
Thiazolidinedione			0.174
No (*n* = 724,992)	1.74	1.25 (1.18–1.31)	
Yes (*n* = 52,200)	2.73	1.39 (1.2–1.61)	
DPP-4 inhibitor			0.182
No (*n* = 744,888)	1.80	1.25 (1.19–1.31)	
Yes (*n* = 32,304)	2.31	1.45 (1.17–1.78)	
α-Glucosidase inhibitor			0.003
No (*n* = 683,859)	1.42	1.2 (1.13–1.27)	
Yes (*n* = 93,333)	4.20	1.4 (1.29–1.53)	
Insulin			0.001
No (*n* = 728,042)	1.14	1.19 (1.12–1.26)	
Yes (*n* = 49,150)	8.38	1.42 (1.31–1.54)	

Adjusted for age, sex, body mass index, smoking, alcohol drinking, exercise, presence of dyslipidemia, hypertension, chronic kidney disease, low-income status, duration of diabetes as continuous variable, the number of classes of oral glucose-lowering medicine, presence of prescription history of insulin, mean fasting, and the number of exams. Each variable used to stratify the participants was excluded from the adjustment.

## Data Availability

The data that support the findings of this study are available from the National Health Insurance Corporation, but restrictions apply to the availability of these data, which were used under license for the current study, and thus are not publicly available. Data are, however, available from the authors upon reasonable request and with permission of the National Health Insurance Corporation.

## Supplementary Materials

The following are available online at https://www.mdpi.com/article/10.3390/jcm10245948/s1, Figure S1: Selection of study subjects. Figure S2: Study design showing the period estimating glucose variability and the risk of incident end-stage kidney disease (ESKD). Table S1: Hazard ratios (HRs) and 95% confidence intervals (CIs) for the incidence of end-stage kidney disease by deciles of fasting glucose variability. Table S2: Hazard ratios (HRs) and 95% confidence intervals (CIs) for the incidence of end-stage kidney disease by quartiles of fasting glucose variability, assessed by standard deviation, coefficient of variation, and average real variability. Table S3: Hazard ratios and 95% confidence intervals for the incidence of end-stage of renal disease according to baseline fasting glucose concentration.
