# Supplementary material for: Fasting Glucose Variability as a Risk Indicator for End-Stage Kidney Disease in Patients with Diabetes: A Nationwide Population-Based Study
Source: J Clin Med. 2021 Dec 18;10(24):5948. doi: 10.3390/jcm10245948 (PMC8705330; doi:10.3390/jcm10245948)
Supplement: Supplementary file 1 [file jcm-10-05948-s001.zip › jcm-1481099-supplementary.pdf]

Supplementary Table S1. Hazard ratios (HRs) and 95% confidence intervals (CIs) for the incidence of end-stage kidney disease by deciles of fasting glucose variability.

	Events (n)	Follow-up duration (Person-years)	Incidence rate (per 1000 person-years)	Multivariate-adjusted HR (95% CI)
D1 (N = 77,719)	549	589,756.6	0.93	1 (Ref.)
D2 (N = 77,720)	562	591,905.4	0.95	1.04 (0.92–1.17)
D3 (N = 77,718)	577	593,276.2	0.97	1.01 (0.9–1.14)
D4 (N = 77,716)	584	593,566.6	0.98	1.0 (0.9–1.13)
D5 (N = 77,720)	627	593,598.4	1.06	1.03 (0.91–1.15)
D6 (N = 77,719)	663	593,304.4	1.12	1.06 (0.94–1.18)
D7 (N = 77,723)	705	593,048.9	1.19	1.06 (0.95–1.19)
D8 (N = 77,719)	767	592,077.8	1.30	1.1 (0.99–1.23)
D9 (N = 77,719)	925	589,935.8	1.57	1.22 (1.09–1.35)
D10 (N = 77,719)	1,331	582,716.8	2.28	1.41 (1.28–1.56)
P for trend				<0.001

a D1:0-7.6; D2:7.7–11.1; D3:11.2–14.2; D4:14.3–17.3; D5:17.4–20.5; D6:20.6–24.2; D7:23.3–28.6; D8:28.7–34.3; D9:34.4–43.1; D10: ≥43.2.

Adjusted for age, sex, body mass index, smoking, alcohol drinking, exercise, presence of dyslipidemia, hypertension, chronic kidney disease, low-income status, duration of diabetes as continuous variable, the number of classes of oral glucose-lowering medicine, presence of prescription history of insulin, mean fasting, and the number of exams.

Supplementary Table S2. Hazard ratios (HRs) and 95% confidence intervals (CIs) for the incidence of end-stage kidney disease by quartiles of fasting glucose variability, assessed by standard deviation, coefficient of variation, and average real variability.

	Events (n)	Incidence rate (per 1000 person-years)	Multivariate-adjusted HR (95% CI)
**FG SD Quartiles**[a]			
Q1 (n = 193,996)	1,115	0.76	1 (Ref.)
Q2 (n = 194,695)	1,227	0.82	0.92 (0.85–0.99)
Q3 (n = 194,206)	1,439	0.97	0.89 (0.82–0.96)
Q4 (n = 194,295)	3,509	2.40	1.17 (1.08–1.26)
**FG CV Quartiles**[b]			
Q1 (n = 194,297)	1,215	0.82	1 (Ref.)
Q2 (n = 194,298)	1,369	0.92	0.99 (0.92–1.08)
Q3 (n = 194,299)	1,631	1.10	0.99 (0.91–1.06)
Q4 (n = 194,298)	3,075	2.10	1.22 (1.14–1.31)
**FG ARV Quartiles**[c]			
Q1 (n = 193,366)	1,073	0.73	1 (Ref.)
Q2 (n = 192,075)	1,153	0.78	0.92 (0.85–1.01)
Q3 (n = 198,086)	1,537	1.02	0.94 (0.87–1.02)
Q4 (n = 193,665)	3,527	2.42	1.19 (1.1–1.29)

[a] Q1: 0–10.7; Q2: 10.8–18.8; Q3: 18.9–31.8; Q4: ≥31.9.

[b] Q1: 0–9.4; Q2: 9.5–15.6; Q3: 15.7–24.3; Q4: ≥24.4.

[c] Q1: 0–12.4; Q2: 12.5–21.4; Q3: 21.5–37.2; Q4: ≥37.3.

Adjusted for age, sex, body mass index, smoking, alcohol drinking, exercise, presence of dyslipidemia, hypertension, chronic kidney disease, low-income status, duration of diabetes as continuous variable, the number of classes of oral glucose-lowering medicine, presence of prescription history of insulin, mean fasting, and the number of exams.

Supplementary Table S3. Hazard ratios and 95% confidence intervals for the incidence of end-stage of renal disease according to baseline fasting glucose concentration

	Events (n)	Follow-up duration (person-years)	Incidence rate (per 1000 person-years)	Age- and sex-adjusted HR (95% CI)	Multivariate-adjusted HR (95% CI)	
					Model 1	Model 2
**End-stage of renal disease**						
<80 mg/dL (n = 20,822)	425	153871.8	2.76	2.39 (2.15–2.67)	2.07 (1.86–2.31)	1.48 (1.32–1.65)
80-99 mg/dL (n = 122,570)	1221	921938.2	1.32	1.17 (1.08–1.27)	1.16 (1.07–1.25)	1.24 (1.15–1.34)
100-119 mg/dL (n = 158,072)	1368	1199716.1	1.14	1(Ref.)	1(Ref.)	1(Ref.)
120-139 mg/dL (n = 216,789)	1231	1664240.4	0.74	0.69 (0.64–0.74)	0.75 (0.7–0.81)	0.77 (0.71–0.83)
140-159 mg/dL (n = 116,220)	823	891204.3	0.92	0.87 (0.79–0.95)	0.95 (0.87–1.03)	0.82 (0.75–0.89)
160-179 mg/dL (n = 55,151)	522	420842.0	1.24	1.18 (1.06–1.3)	1.27 (1.15–1.41)	0.93 (0.84–1.03)
≥180 mg/dL (n = 87,568)	1700	661374.1	2.57	2.56 (2.38–2.75)	2.65 (2.47–2.85)	1.64 (1.52–1.77)

Model 1 is adjusted for age, sex, body mass index, smoking, alcohol drinking, exercise, presence of chronic kidney disease, dyslipidemia, and hypertension, and low-income status.

Model 2 is the same as model 1, plus an adjustment for duration of diabetes as continuous variable, the number of classes of oral glucose-lowering medicine, the presence of prescription history of insulin, and the number of exams.

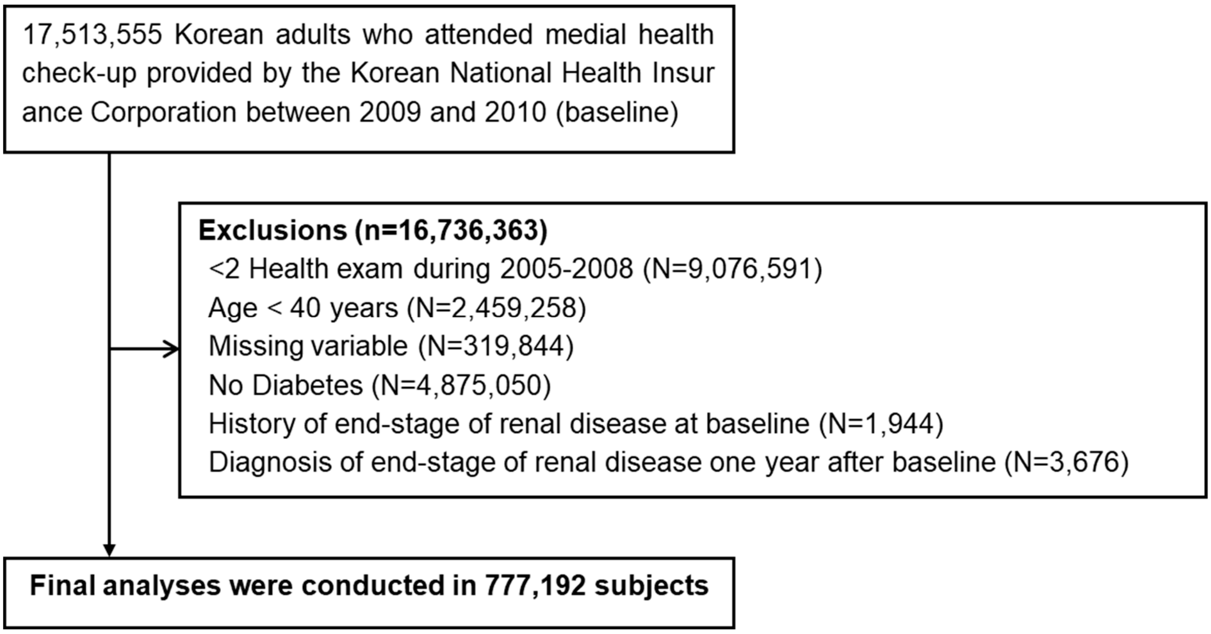

Supplementary Figure S1. Selection of study subjects.

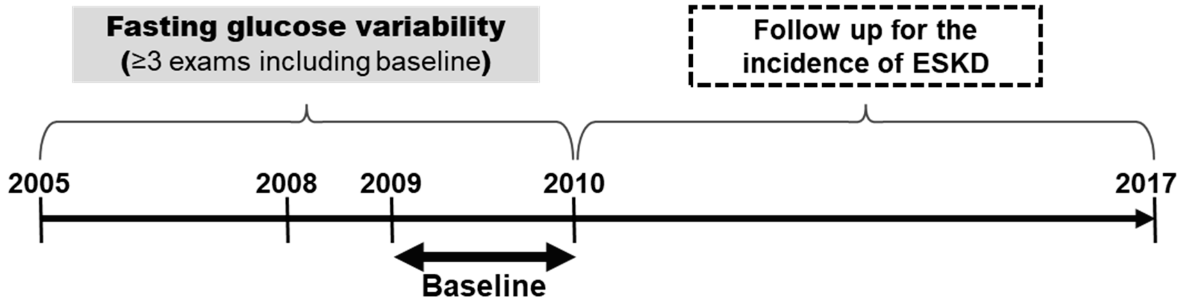

Supplementary Figure S2. Study design showing the period estimating glucose variability and the risk of incident end-stage kidney disease (ESKD).
